# Topically Applied Etamsylate: A New Orphan Drug for HHT-Derived Epistaxis (Antiangiogenesis through FGF Pathway Inhibition)

**DOI:** 10.1055/s-0039-1693710

**Published:** 2019-07-26

**Authors:** Virginia Albiñana, Guillermo Giménez-Gallego, Angela García-Mato, Patricia Palacios, Lucia Recio-Poveda, Angel-M Cuesta, José-Luis Patier, Luisa-María Botella

**Affiliations:** 1 Molecular Biomedicine Department, Centro de Investigaciones Biológicas, CSIC, Madrid, Spain; 2 Centro de Investigación Biomédica en Red, CIBERER, Instituto de Salud Carlos III, Madrid, Spain; 3 Department of Internal Medicine, University Hospital Ramón y Cajal; Department of Medicine and Medical Specialities, Faculty of Medicine, University of Alcalá, IRYCIS, Madrid, Spain

**Keywords:** HHT, Endoglin, Alk1, antiangiogenesis, etamsylate, epistaxis, FGF pathway, TGF-β signaling pathway

## Abstract

Hereditary hemorrhagic telangiectasia (HHT) is a vascular dysplasia characterized by recurrent and spontaneous epistaxis (nose bleeds), telangiectases on skin and mucosa, internal organ arteriovenous malformations, and dominant autosomal inheritance. Mutations in *Endoglin* and *ACVRL1* / *ALK1* , genes mainly expressed in endothelium, are responsible in 90% of the cases for the pathology. These genes are involved in the transforming growth factor-β(TGF-β) signaling pathway. Epistaxis remains as one of the most common symptoms impairing the quality of life of patients, becoming life-threatening in some cases. Different strategies have been used to decrease nose bleeds, among them is antiangiogenesis. The two main angiogenic pathways in endothelial cells depend on vascular endothelial growth factor and fibroblast growth factor (FGF). The present work has used etamsylate, the diethylamine salt of the 2,5-dihydroxybenzene sulfonate anion, also known as dobesilate, as a FGF signaling inhibitor. In endothelial cells, in vitro experiments show that etamsylate acts as an antiangiogenic factor, inhibiting wound healing and matrigel tubulogenesis. Moreover, etamsylate decreases phosphorylation of Akt and ERK1/2. A pilot clinical trial (EudraCT: 2016–003982–24) was performed with 12 HHT patients using a topical spray of etamsylate twice a day for 4 weeks. The epistaxis severity score (HHT-ESS) and other pertinent parameters were registered in the clinical trial. The significant reduction in the ESS scale, together with the lack of significant side effects, allowed the designation of topical etamsylate as a new orphan drug for epistaxis in HHT (EMA/OD/135/18).

## Introduction

Hereditary hemorrhagic telangiectasia (HHT) or Rendu–Osler–Weber syndrome (OMIM # 187300) is a vascular hereditary autosomal-dominant disease associated to epistaxis, telangiectases, gastrointestinal hemorrhages, and arteriovenous malformations (AVMs) in the lung, liver and brain. Prevalence is around 1 in 5,000 to 8,000 according to recent reviews. 1 2 3 4

The disease diagnosis is based on clinical symptoms: the Curaçao criteria. 5 A patient is considered to have HHT if he has, at least, three out of the following four criteria: (1) spontaneous and recurrent epistaxis; (2) multiple telangiectases at characteristic sites (lips, oral cavity, fingers, and nose); (3) visceral lesions (gastrointestinal telangiectases, pulmonary, hepatic, cerebral, or spinal AVMs); or (4) a first-degree relative with HHT according to these criteria. The clinical diagnosis requires then a detailed medical screening.

The penetrance of the disease is variable, depending on age and increasing with it. At 45 years, penetrance is approximately 90%. 6 Since HHT patients may have lung and brain AVMs before the onset of epistaxis and telangiectases, the establishment of an early molecular diagnosis is necessary. These malformations may give rise to life-threatening complications, such as brain ictus, brain infarction, brain abscesses, massive hemoptysis and paralysis. Thus, all relatives of a family diagnosed with HHT should be subjected to a genetic testing.

Two loci are involved by mutation in more than 90% of HHT cases. The first gene identified was *Endoglin* , 7 representing between 39 and 59% of the HHT cases, called type I or HHT1. Shortly after, *ALK1* (also known as *ACVRL1* ) was described, 8 as being involved in 25 to 57% of the HHT cases, called type II or HHT2. In around 2% of all HHT patients, the origin of the disease is a mutation in the *MADH4* gene leading to the combined syndrome of juvenile polyposis and HHT (JPHT). 1 Other loci involved in the origin of the disease in minor cases have been described: a locus, on chromosome 5 linked to HHT in two English cohorts, whose gene is still unidentified. 9 A fourth locus was also reported associated to chromosome 7. 10 Even more recently, mutations in BMP9, a transforming growth factor-β (TGF-β) superfamily ligand of ALK1, have been described as causing HHT5. 11 The common link among all these genes is that they belong to the TGF-β signaling pathway.

Since there is no real cure for HHT, the research is centered in developing safe and efficient treatments to control epistaxis and gastrointestinal bleeding. The therapeutic strategies may be classified in different categories according the way of action.

- Strategies aiming to improve coagulation and stabilizing coagulation versus fibrinolysis. Antifibrinolytics such as tranexamic acid and ε-aminocaproic acid are among the most used drugs in the first line of treatment. 12
- The upregulation of Endoglin or ALK1 protein content at the plasmatic membrane of the endothelial cells to counteract haploinsufficiency in HHT. Drugs such as FK506, raloxifene, and bazedoxifene have been used in this strategy. Raloxifene and bazedoxifene, both selective estrogen receptor modulators (SERMs), were designated orphan drugs for HHT by the European Medicines Agency (EMA) in 2010 and 2014, respectively. 13 14 15
- To decrease or interfere with neoangiogenesis, to avoid the abnormal and excessive dilated and tortuous vessels on the nose mucosa, since HHT is also characterized by a deregulation of the angiogenic process. In this section, antiangiogenic drugs such as bevacizumab (Avastin), β-blockers like propranolol, and the new orphan drug subject of the present article, etamsylate, are included. 12 16 Etamsylate blocks the assembly of the complex that triggers fibroblast growth factor (FGF) signaling at the cell level. 17

FGFs constitute one of the largest families of polypeptide growth factors. Apart from the subset comprising FGF-11 to FGF-14, FGFs exert their diverse biological actions by binding to a series of membrane tyrosine kinase receptors (FGR receptors, FGFRs) that are encoded by four genes. For this reason the FGF family is currently considered to be constituted by 18 members. 18 19 FGFs govern a wide assortment of developmental and physiological processes, as practically all cell lineages derived from the embryonic mesoderm and neuroectoderm are under their control.

Acidic and basic FGFs (aFGF and bFGF, respectively) were the two members of the group first described. They were later renamed FGF-1 and FGF-2. They have very similar biochemical and biological properties and are considered paradigms for the whole family. 20 21 FGFs, at times expressed at very high levels, normally remain trapped in the extracellular matrix, from which they are released by heparanases or other specialized proteins when necessary. Obviously, the subversion of this powerful signaling system and any defects in its tight control, either through uncontrolled synthesis or the continuous mobilization of matrix-bound FGFs, may cause very serious physiological disturbances. 20 22 23 24 25 In addition, it has been shown that different types of stress, such as heat shock, hypoxia, and low serum, induced export of FGF-1. 26 27 28

FGF-1 and FGF-2 were the first two pure polypeptides shown to promote angiogenesis. 29 30 Later on, more than a dozen factors that promote angiogenesis were described, vascular endothelial-cell growth factor (VEGF) is one among them. FGF and VEGF are probably the only growth factors that directly promote the proliferation of endothelial cells. 31 FGFs constitutively cooperate in VEGF-induced angiogenesis and they are responsible for the development of resistance to VEGF-based antiangiogenic treatments. 32 33 Accordingly, FGF inhibition may constitute an interesting new therapeutic approach to treat diseases caused by excess, uncontrolled angiogenesis. 34 Angiogenesis induced by FGFs leads to the formation of quite tortuous blood vessels similar to those observed in tumor angiogenesis, which are, further, quite permeable. 17 29

Further studies later showed that FGFs were early mediators of FGF-driven angiogenesis in vivo and neovascularization, according to the expression profiling of FGF-stimulated microvascular cells. Inflammation elicited by FGF is prone to the consolidation of a positive inflammatory feedback loop, typical of chronic diseases, since it induces the upregulation of the synthesis of COX-2 and phospholipase A2, which reciprocally promote the expression of FGF. 35 36 37

Many of the features summarized in the previous paragraphs suggest that the inhibition of the FGF signaling system could be an adequate target to treat HHT.

FGF signaling can be inhibited in vitro and in vivo with synthetic compounds. One of these products is the anion 2,5-dihydroxybenzene sulfonate (2,5,DHBS), also known as dobesilate. This compound is commercially available as tablets of its calcium and N-ethylene ethanamine salts (Doxium and Dicynone, respectively). In the case of the second salt (Dicynone or etamsylate), the drug is also available as an injectable solution, which was the pharmaceutical form employed, as spray, in this assay. These drugs have been used, for many years, to treat diabetic retinopathy and chronic venous insufficiency with no reported side effects, although with no clear, statistically significant positive results. 17 38 39 40

No molecular basis accounting for biological activities has been clearly demonstrated until recent years when it was shown that dobesilate interacts with FGF, reducing considerably the affinity by its receptor. Dobesilate belongs to a new class of in vivo FGF inhibitors, headed by gentisic acid, a compound associated with plant defense and a metabolite of aspirin. Dobesilate is the most active member of this family of FGF inhibitors. 17

Direct interaction of dobesilate with FGFs has been thoroughly characterized even at the atomic level. It has been shown that this interaction distorts the interface region of the cellular receptor that detects and triggers the characteristic cell responses evoked by FGF. It has also been extensively shown that dobesilate inhibits, consequently, FGF-dependent cell migration, angiogenesis (in vitro and in vivo), and cell growth. Inhibition by dobesilate of the intracellular signaling cascade triggered by FGF has been also properly characterized. 17 41 42

In HHT, epistaxes are due to an excessive and abnormal vascularization of nasal mucosa. All the pathophysiological consequences of the abnormal high levels of FGF and the mechanisms involved in the generation of such excesses, summarized above, seem to provide a good rationale for an in vivo clinical trial aimed to explore whether dobesilate could be used to treat the abnormal angiogenesis and the subsequent epistaxis, associated with HHT. The trial is registered at AEMPS as “EudraCT: 2016-003982-24.”

## Material and Methods

### Cell Culture

Human microvascular endothelial cell line-1 (HMEC-1) was grown in MCDB131 medium (Gibco) supplemented with EGF (1 ng/mL; Promega) and hydrocortisone (1 ng/mL; Sigma). On the other hand, human umbilical vein endothelial cells (HUVECs) were grown on EBM-2 medium (Lonza), supplemented with SingleQuots (Lonza) endothelial growth medium-2 (EGM-2). All culture media were supplemented with 10% of FBS (Gibco), 2 mM of L-Glutamine and 100 units/mL of penicillin/estreptomicine (Gibco). Blood outgrowth endothelial cells (BOECs) from healthy and HHT donor primary cultures were grown in EGM-2 (Lonza), supplemented with 20% FBS, following the reported protocol. 43 44 Treatments in the absence or presence of FGF were performed in medium EGM with 2% FBS. BOECs from passages 4 and 5, three control groups, and three different HHT donors were used in the experiments.

### Etamsylate Treatment

The etamsylate for in vitro experiments (Alicon Pharmaceuticals) was used at 10, 20, 50, and 100 μM. The length of the treatments was between 24 and 48 hours depending on the experiment.

### Viability Assay

The viability of HUVEC and BOEC cells was measured after 48 hours of treatment at different doses of etamsylate, using CellTiter-Glo Luminescent Cell Viability Assay (Promega), a homogeneous quantitative method to determine the number of viable cells in a culture based on quantitation of the intracellular adenosine triphosphate (ATP) presence, which indicates metabolically active cells. Briefly, 5 × 10 ^3^ cells were seeded in 96-well plates and cultured in 100 μL with increasing doses (0–100 μM) of etamsylate for 48 hours. Then, 100 μL/well of CellTiter-Glo reagent (Lysis buffer, Ultra-Glo Recombinant Luciferase, Luciferin, and Mg ^2+^ ) was added and gently mixed for 15 minutes at room temperature. Next, luminescence was measured using a Glomax Multidetection system (Promega).

### Gene Expression Analysis at the RNA Level

Cells were treated with etamsylate for 24 hours, collected by centrifugation at 1,250 rpm for 5 minutes, and RNA was extracted using the commercial kit NucleoSpin RNA (Macherey-Nagel) following manufacturer instructions. RNA was retro-transcribed from 0.5 to 1 μg total RNA by the commercial kit High Capacity cDNA Reverse Transcription (Applied Biosystems), using random primers in a final volume of 20 μL. cDNA was used as a template for real-time polymerase chain reaction (PCR) using iQTM SYBR Green Supermix (BioRad). Ribosomal 18S RNA was used as a housekeeping control.

The sequences of the primers were:

***ENG*** *: Fwd 5′* AGCCTCAGCCCACAAGT 3′; *Rev 5′* GTCACCTCGTCCCTCTCG 3′
***ACVRL1/ALK1*** *: Fwd 5′* ATCTGAGCAGGGCGACAC 3′; *Rev 5′* ACTCCCTGTGGTGCAGTCA 3′
***18S*** *: Fwd 5′* CTCAACACGGGAAACCTCAC *3′; Rev 5′* CGCTCCACCAACTAAGAACG 3′

### Gene Expression Analysis of Proteins by Western Blotting and ELISA

After 48 hours of etamsylate treatment at the different concentrations, cells were lysed in TNE 1X (Tris 10 mM, pH = 8; NaCl 150 mM, and EDTA 0.1 nM, pH = 8), Tritón X-100 (0.5%), with phosphatases and proteases inhibitors, from Sigma, and 5 μM Lactacystin proteasomal inhibitor (Sigma). Protein lysates were separated by SDS-PAGE electrophoresis in 10% polyacrylamide gels, in Mini-Protean III equipment (BioRad). Proteins were transferred to nitrocellulose membranes, blocked, and incubated with the different primary antibodies and secondary horseradish peroxidase (HRP) conjugated antibodies according to the table. Antibodies p-Akt, Akt, and p-ERK1/2 were antihuman raised in rabbit from Cell Signaling, while Erk1/2 and tubulin were mouse antihuman (Cell Signaling and Sigma-Aldrich, respectively).

Determination of VEGF and FGF levels in human plasma samples was made by ELISA. Quantikine kits were obtained from R&D Systems, Minneapolis, Minnesota, United States.

### Angiogenesis Two-Dimensional Assays

Wound healing assays were performed in confluent monolayers of endothelial cells pretreated for 24 hours with different etamsylate doses, before scratching the monolayers with the tip of a micropipette. After that, cells were washed in PBS, and incubated with fresh culture medium with the corresponding pretreatment dose. The migration of cells on the “wound” edges was followed at different times and recorded by photography, to measure the relative mobility of cells by studying the width between the advancing edges. The tubulogenesis assay with endothelial cells was made on Matrigel (BD). Cells were pretreated for 24 hours with the different etamsylate doses. A total of 80,000 to 90,000 cells were plated on individual P-24 Matrigel-coated wells. Photographs were taken every 2 hours. The average of the widths in three to five different wells was used to document the mobility at different times.

### Fibrin Gel Beads Three-Dimensional (3D) Angiogenesis Assays

Cytodex beads (100 µL) were incubated for 4 hours at 37°C with 10 ^6^ HUVECs in 1.5 mL of EGM-2 medium. These HUVEC-coated beads were next transferred to a 15-mL Falcon tube containing fibrinogen (2.0 mg/mL) and aprotinin (0.15 units/mL) in a ratio of 500 beads/mL. Next, 0.5 mL of beads solution was plated in each of the wells of a P-24 plate, previously treated with 0.625 units of thrombin per well. Then etamsylate was added in different concentrations to the P-24 wells, and photographs under the microscope were taken in the following days to observe interconnection between the HUVEC-covering contiguous beads.

### Clinical Trial: Protocol Code: HHT-HOPE-2016

#### EudraCT No.: 2016–003982–24

- **Treatment:** To prepare the nose pulverizer, a total of 4 mL of etamsylate from injection vials (Dicynone) was used to fill a sterile amber glass vial with an adjustable top for spray dosification. The regimen of etamsylate (Dicynone) dosage used in this trial for epistaxis in HHT was two spray “puffs” per day in each nostril, spaced 12 hours, equivalent to 200 μL per day of a 125 mg/mL solution, i.e., 25 mg.
  A total of 12 patients were enrolled in the study. Each patient was subjected to monitoring for up to 4 weeks. The study started in May 2017 and was ended in March 2018.
- **Inclusion criteria:** The target population for the clinical trial was constituted by HHT patients diagnosed by the presence of three or four Curaçao criteria. In addition, all of them had also the genetic diagnosis test made with known mutations. They had significant epistaxis in all cases.
- **Explicit inclusion criteria from** **clinicaltrialsregister.eu** **:** Adult patients (18 years or more) of both sexes, diagnosis of HHT, and with high propensity for nosebleeds. Patients with ability and willingness to follow the study protocol and give their informed consent (signed and dated), agreeing to participate voluntarily in the study.
- **Explicit exclusion criteria from** **clinicaltrialsregister.eu** **:** Do not sign the informed consent to participate in the study after being informed by the investigator on the target, the course, and potential risks of the study consent. Patients who cannot meet the requirements of the study or in the investigator's opinion should not participate in the study. Patients with concomitant diseases, according to the researcher may influence (by the disease itself and/or its treatment) in the development, evolution, or valuation of HHT. Patients who have received anti-inflammatory treatment in the last month. Patients in whom the use of etamsylate is contraindicated. Pregnant or breast-feeding.

#### End-Points of the Clinical Trial

- The main end point of the clinical trial was a reduction in the epistaxis severity score (ESS) 45 after 4 weeks of treatment, in the absence of adverse events.
- Secondary end-points: evaluate the effect of etamsylate to improve anemic situations (measured by hemoglobin and hematocrit blood test) and the incidence of the treatment on the quality of life of patients.

#### Epistaxis Severity Score

The ESS is recommended as a standard consensus validated method to measure epistaxis in HHT, and was used in the present study. The score consists of six questions of multiple choices and each question has a correction factor so that the final score may result from 0 to 10. Epistaxis may be considered from none to severe.

### Summary

In vivo clinical trial registered in the AEMPS as N° de EudraCT: 2016–003982–24.

National competent authority: Spain—AEMPS.

### Statistics

Student *t* -tests were made. The different levels of significance were as follows: * *p* < 0.05, ** *p* < 0.01, and *** *p* < 0.001. Data were represented as the mean ± SD (standard deviation). When making statistics of VEGF and FGF results comparing pre- and posttreatment, the comparison was made with the Friedman's test, for no-parametric paired values. Experiments were performed in triplicate and repeated at least three independent times. The results shown are representative.

## Results

### Etamsylate Effect on In Vitro Two-Dimensional Angiogenesis

#### Etamsylate Inhibits Migration and Tubulogenesis on Matrigel in Endothelial Cells

Two different functional assays were performed to evaluate the etamsylate effect on endothelial cell angiogenesis: the wound healing assay and the tubulogenesis on Matrigel.

**Fig. 1:**
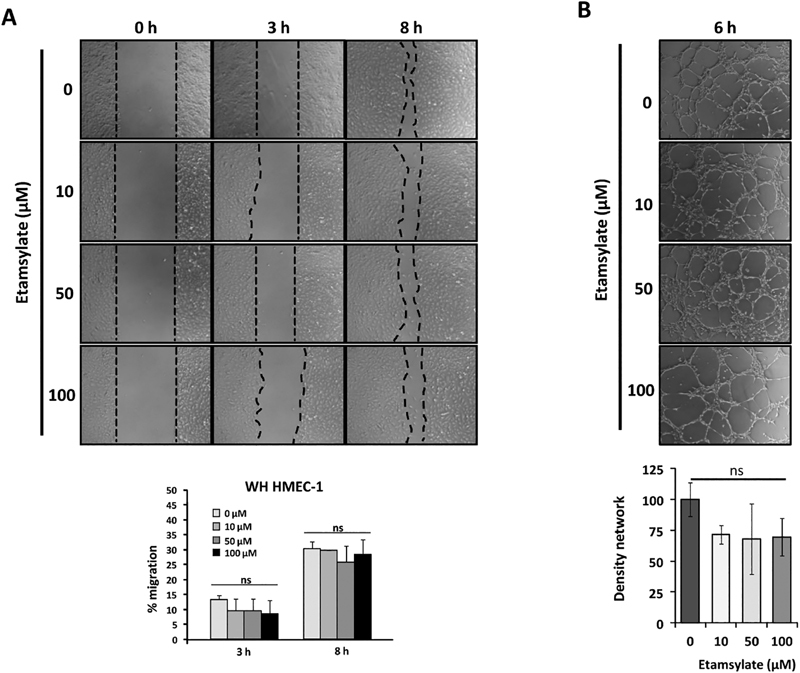
Effect of etamsylate on angiogenesis in HMEC-1 cells. ( **A** ) Wound healing assay. In the upper part, representative images of the different experiments and replicas are shown. In the lower part, the percentage of migration in time is shown at different doses. ( **B** ) Tubulogenesis assay. In the upper part, representative images are shown. In the lower part, the tube densities per field at different doses compared with the untreated cells are shown. Mean with the standard deviation is represented normalized versus control. Statistical significance was studied using Student's *t* -test. No significant differences were found in this case (ns).

As shown in Fig. 1A , there were no significant differences in the migration of HMEC-1 cells treated with etamsylate or not.

Concerning the tubulogenesis assay, etamsylate-treated HMEC-1 cells showed a decreased amount of closed cells on Matrigel compared with control (untreated). On the other hand, the morphology of the closed network was thinner and bigger in treated cells than in untreated ones ( Fig. 1B ). As in the previous cases, the differences were not statistically significant.

**Fig. 2:**
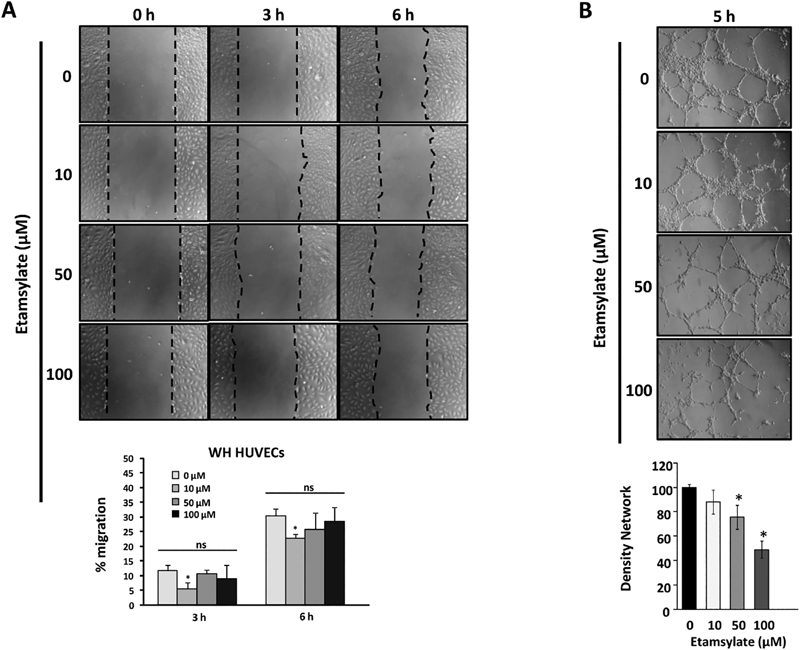
Effect of etamsylate on angiogenesis in HUVECs. ( **A** ) Wound-healing assay. In the representative images shown, the cell migration after etamsylate administration is observed. In the lower part, the percentage of wound healing (migration) in time is represented. The mean with SD is shown. Values are normalized with control. Statistical significance was studied using Student's *t* -test. * *p* < 0.05. ( **B** ) Tubulogenesis assay. Representative images are shown, demonstrating a lower amount of tubes in the treated cells compared with untreated cells. The different tube densities are shown in a representative experiment. The mean with SD is shown. Values are normalized with control. Statistical significance was studied using Student's *t* -test. * *p* < 0.5. HUVECs, human umbilical vein endothelial cells.

Cell migration and tubulogenesis on Matrigel were also tested in HUVECs. Cells treated with etamsylate were significantly delayed in closing the discontinuity (wound) at 10 μM ( Fig. 2A ; *p* = 0.021 and *p* = 0,026, after 3 and 6 hours, respectively). In tubulogenesis, the amount of tubes formed on Matrigel in etamsylate HUVECs is significantly lower, decreasing by 24% at 50 μM (* *p* = 0.046) and by 51% at 100 μM (*** *p* = 0.0001). Moreover, the tubes formed under etamsylate treatment are thinner and the closed cells are larger than the control ones ( Fig. 2B ).

**Fig. 3:**
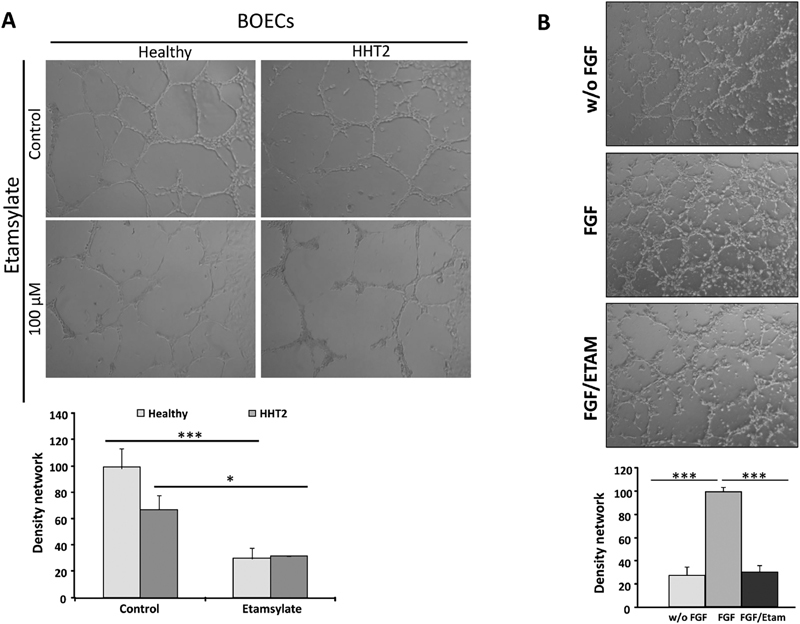
( **A** ) Effect of etamsylate on tubulogenesis assay in BOECs. Representative images demonstrate a lower amount of tubes in the treated cells. The different tube densities are shown in the graph. The number of closed tubules was counted in five different fields and the mean was calculated. The mean with SD is shown. Statistical significance was studied using Student's *t* -test (healthy: *p* < 0.002; HHT: *p* < 0.023). ( **B** ) Tubulogenesis assay FGF vs. FGF/etamsylate. HHT BOECs were treated with or without FGF and etamsylate. The number of closed tubules was counted in five different fields and the mean was calculated. The images and graph show a significant decrease in the number of closed tubes when cells are without FGF (*** *p* < 2.6 × 10 ^−6^ ), and when etamsylate is added in the presence of FGF (FGF/etamsylate; *** *p* < 1.1 × 10 ^−6^ ) in comparison with FGF-treated cells (FGF). BOECs, blood outgrowth endothelial cells; FGF, fibroblast growth factor; HHT, hereditary hemorrhagic telangiectasia.

Tubulogenesis assay with healthy and HHT2 BOECs is shown in Fig. 3A . As in previous cases, the tubes formed under etamsylate treatment are significantly decreased and less closed (healthy: *** *p* = 0.002; HHT: * *p* = 0.023)

To demonstrate that etamsylate blocks the FGF pathway, HHT BOECs were cultivated in the presence and absence of FGF, and the etamsylate effect was evaluated. The tubulogenesis assay showed a significant decrease in the number of closed tubes when cells are without FGF (w/o FGF; *** *p* = 2.6 × 10 ^−6^ ), and when etamsylate is added in the presence of FGF (FGF/etamsylate; *** *p* = 1.1 × 10 ^−6^ ), in comparison with FGF-treated cells (FGF; Fig. 3B ).

**Fig. 4:**
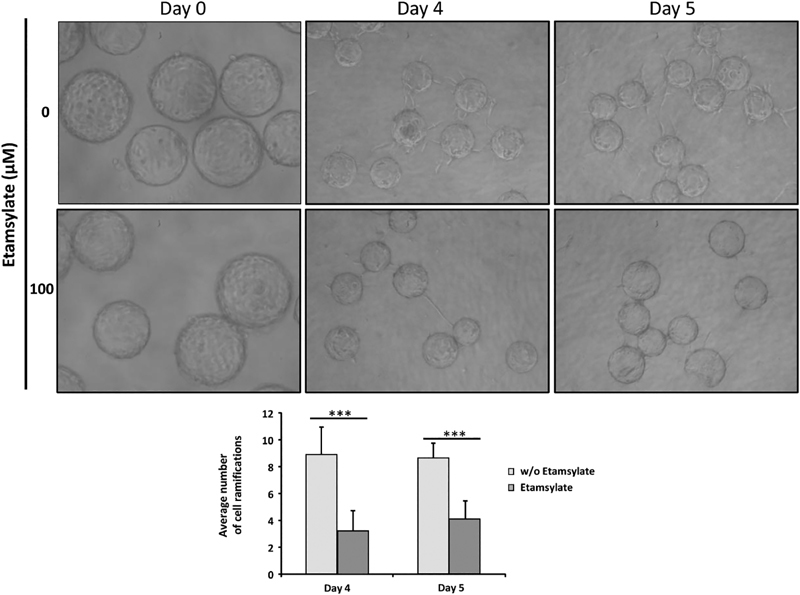
3D angiogenesis. HUVEC-coated beads were cultured in the presence or absence of etamsylate during 5 days. When etamsylate is present, fewer bonds can be observed between neighboring beads forming a network, as it occurs in control conditions. There was a significantly higher number of ramifications in control (untreated cells) compared with etamsylate-treated cells, as the graph shows (day 4, *** *p* < 6.5 × 10 ^−4^ ; day 5, *** *p* = 8.34 × 10 ^−5^ ). HUVECs, human umbilical vein endothelial cells.

Angiogenesis 3D: An optimized fibrin gel beads assay was made to study 3D angiogenesis. Results were followed up to 5 days. The pictures show those days when the angiogenesis process was observed. Later on, the connections between the beads start to lose. There was a significantly higher number of ramifications appearing in the control (untreated cells) compared with the ones treated with etamsylate (day 4: *** *p* < 6.5 × 10 ^−4^ ; day 5: *** *p* = 8.34 × 10 ^−5^ ) as the graph shows ( Fig. 4 ).

## Effect of Etamsylate on Cell Viability of Endothelial Cells

Etamsylate does not significantly affect cell endothelial viability. This fact was proved in HUVECs and HHT BOECs treated in a range from 0 to 100 µM of etamsylate. However, viability was quantified by the amount of intracellular ATP.

**Fig. 5:**
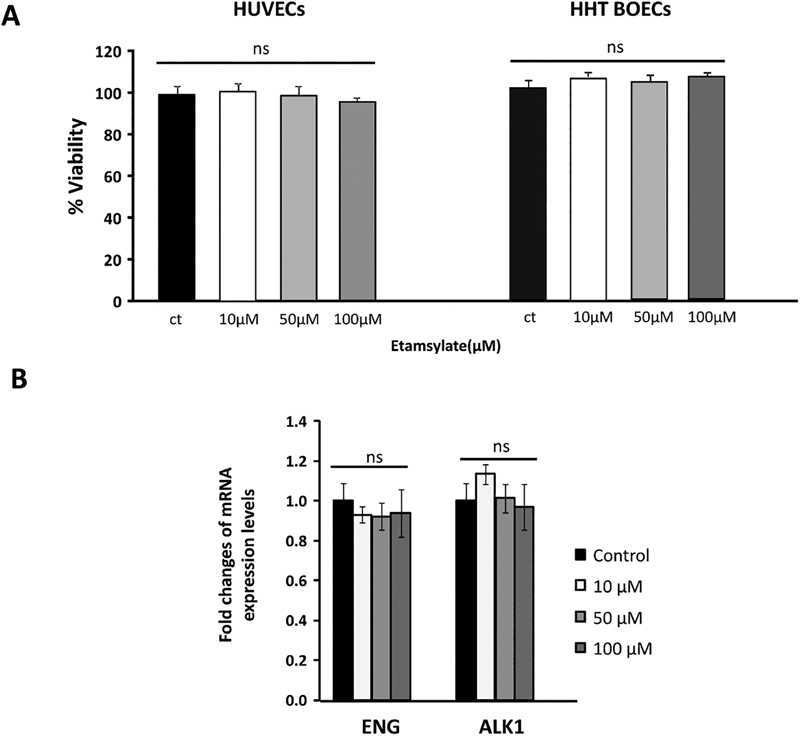
Effect of etamsylate on viability and Endoglin and ALK1 RNA expression. ( **A** ) Viability of HUVECs and BOECs cells in the 0 to 100 μM range of etamsylate treatment is not affected. ( **B** ) RNA expression of ENG and ALK1 after treatment with different doses of etamsylate in HUVECs. RNA levels of *ACVRL1/ALK1* and *Endoglin* were determined by RT-qPCR in HUVECs, after etamsylate treatments (range: 10–100 μM), and normalized with the untreated controls. Statistical significance was studied using Student's *t* -test. No significant difference was found. BOECs, blood outgrowth endothelial cells; HUVECs, human umbilical vein endothelial cells.

As shown in Fig. 5A , there are no significant differences between viabilities in the range etamsylate was used compared with the untreated control.

Thus, as conclusion, we can say that in the 0 to 100 μM range of etamsylate treatment, viability of endothelial cells is not affected.

## Expression of ALK1 and Endoglin after Etamsylate Treatment

Next, it was important to test the impact of different amounts of etamsylate, to elucidate its putative effect on the amount of Endoglin and ALK1 transcripts or to discard a direct effect on them. RNA levels of *ACVRL1/ALK1* and *Endoglin* were determined by RT-qPCR in HUVECs, after etamsylate treatments (range: 10–100 μM) and normalized with the untreated controls. The results are shown in Fig. 5B . No significant differences were found among the different treatments in relation to untreated cells. Therefore, etamsylate does not affect the expression levels of *ACVRL1/ALK1* and *Endoglin* , target genes in HHT. Samples treated in parallel with the same cells were subjected to Western blot to see whether etamsylate was really affecting the FGF signaling cascade, and the results are shown in the next section.

## Etamsylate Inhibition of FGF/FGFR Signaling Decreases Phosphorylation Levels of *AKT* and *Erk1/2*

**Fig. 6:**
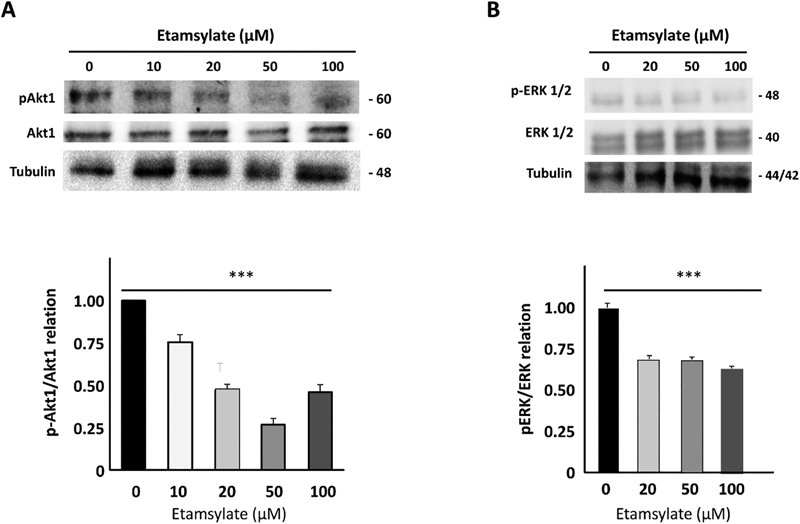
Analysis of Akt and Erk1 phosphorylation after etamsylate treatment. ( **A** ) Analysis of pAkt1, Akt1, and tubulin in cell lysates in HUVECs. A representative gel of three experiments performed is shown. Bands were quantified by Adobe Photoshop CS. Relative levels of pAkt1 normalized versus total Akt1. A decrease of the amount of phosphorylated protein is apparent compared with control. ( **B** ) Relative levels of pERK2 normalized versus total erk. pERK2 levels decrease when cells were treated with the highest doses of etamsylate (50–100 μM) compared with the control. HUVECs, human umbilical vein endothelial cells.

In endothelial cells FGF triggers cell signaling through a cascade involving Akt. The inhibition of FGF/FGFR-mediated signaling by etamsylate in endothelial cells was explored by Western blot. Out of the three Akt kinases (Akt1, Akt2, and Akt3), Akt1 phosphorylation was studied ( Fig. 6A ) since it is the predominant isoform in endothelial cells. 46 In Fig. 6A , the relative values of pAkt1 versus total Akt1 at different etamsylate concentrations are shown. A decrease of around 50% in the phosphorylated protein is observed from etamsylate concentrations above 20 μM.

Moreover, the activity of ERK1 and ERK2 kinases was studied since FGF has been shown to induce these stress MAPK kinases. 47 48 As shown in Fig. 6B , the levels of pErk1/2 decrease after treatment with the two higher concentrations of etamsylate (50 and 100 μM). The levels of pErk were normalized with total Erk and tubulin ( Fig. 6B ). Normalization was checked by both procedures, getting the same results.

## Results of the Clinical Trial

The results of the clinical trial involving 12 patients are presented in Table 1 . This is an initial cohort of 12 patients with clinical and genetic diagnoses of HHT (6 ENG and 6 ACVRL1). Average age 52 (interval 23 and 72); 7 women and 5 men; with different values according to the ESS; range [7.3–2.4]. At the beginning of the trial, 30% of the patients had Hb < 12 gr/dL, and 50% ferropenia with ferritin < 15 ng/dL. In no patient was there a concomitant deficit of vitamin B12 or folic acid. All patients had a minimum follow-up of 1 year prior to inclusion in the trial. Their usual management included daily hygiene and hydration of the nasal mucosa in 100% of cases and oral and/or periodic intravenous iron in 80%. Two patients with arterial hypertension had modified their treatment by adding oral propranolol at a dose of 60 mg daily. No patient was on topical propranolol. One patient with postmenopausal osteoporosis was on raloxifene at a dose of 60 mg daily. A patient with atrial fibrillation was on anticoagulant treatment with acenocumarol. A patient after finishing the trial declared weekly anabolic injections as part of his personal training. No patient had undergone nasolabial telangiectasia sclerosis since 6 months prior to or during the trial. Data concerning the type of HHT (HHT1 or HHT2), hemoglobin, iron, and ferritin levels, as well as the HHT-ESS normalized score, before and after the clinical trial, are shown for each patient. The patient's diary was also obtained during the 28 days of study. The analysis of the patient's diary was used to assess compliance by patients and the occurrence of side effects. It was another way to measure the severity of the epistaxis, and the register of the diary was in agreement with the ESS.

**Table 1:** Clinical results before and after etamsylate treatment

			Before treatment				After treatment			
Patient	Mutation	Sex	Hb	Fe	ferritin	ESS _b_	Hb	Fe	ferritin	ESS _a_
1	*ACVRL1/Alk1*	F	12.3	51	11	7.36	11.2	33	7	4.42
2	*ACVRL1/Alk1*	F	13.3	86	64	3.15	13.5	34	13	2.43
3	*Endoglin*	F	10.9	25	14	4.57	10.8	22	61	1.74
4	*ACVRL1/Alk1*	F	10.4	24	20	5.07	11.2	24	12	3.15
**5**	*ACVRL1/Alk1*	F	13.1	78	23	3.33	12.6	87	22	3.33
6	*Endoglin*	M	15.3	47	28	3.84	15.7	66	31	2.43
7	*Endoglin*	M	16.1	56	18	3.33	16.4	97	24	2.43
8	*Endoglin*	F	12.5	21	3	5.25	–	25	2	3.44
9	*Endoglin*	M	16.1	35	49	2.43	16.3	101	92	1.92
10	*ACVRL1/Alk1*	M	12.7	28	5	3.84	12.4	32	6	2.43
11	*Endoglin*	F	15.-6	74	41	3.33	15.1	70	57	2.43
12	*ACVRL1/Alk1*	M	16.8	123	95	2.43	16.7	98	76	3.33

Abbreviations: ESS, epistaxis severity score (subscripts “a” and “b” indicate after and before treatment); F, female; Fe, iron; Hb, hemoglobin; M, male.

**Fig. 7:**
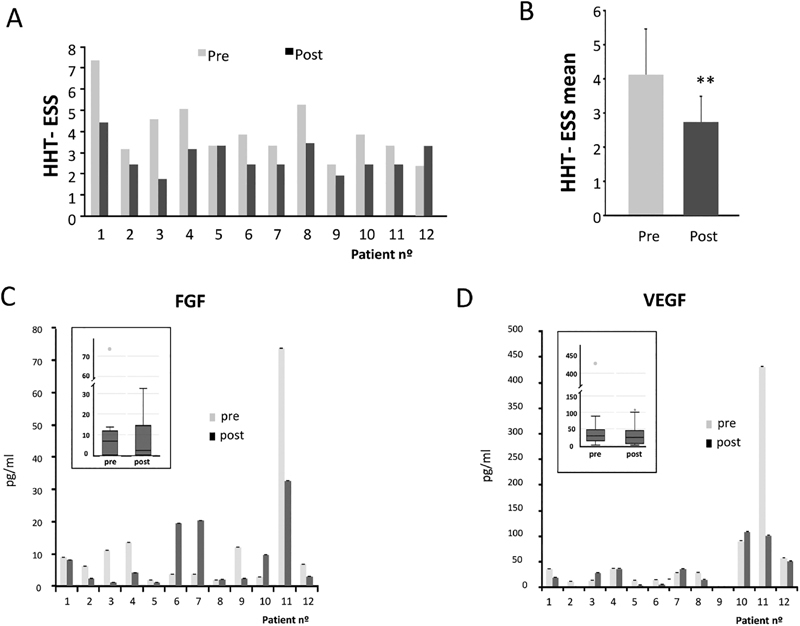
Levels of epistaxis severity score (ESS). ( **A** ) HHT-ESS of the 12 patients before and after the treatment. ( **B** ) HHT-ESS mean of pre- and posttreatment levels of the patients. A Student's *t* -test was made to compare pre- and posttreatment HHT-ESS values. Differences were significant at *p* < 0.01. ( **C** and **D** ) Analysis of FGF and VEGF, as biomarkers related to angiogenesis and bleeding. The graph shows pre- and posttreatment results. FGF, fibroblast growth factor; HHT, hereditary hemorrhagic telangiectasia; VEGF, vascular endothelial growth factor.

Out of 12 patients, 10 patients were responsive. Six patients were found to have at least 1 point lower ESS ( Fig. 7A ). Four patients were found to have decreased ESS between 0.51 and 0.9. One patient's ESS was increased by 0.9 and unchanged in one. Comparing the mean ESS of the 12 patients before and after 1 month of treatment: 3.99 versus 2.79, the decrease was 1.20 in the ESS score, being highly significant with a *p* < 0.001 (***).

Of note, patient 3 was not changing and patient 12, computed for the ESS mean comparisons, was worsening, increasing by almost 1 in the ESS after 1 month. This patient was finally withdrawn from the clinical trial due to the use of anabolic injections to build muscle mass. Although the exclusion criteria did not specifically consider the treatment with anabolizers, it was written that: patients with concomitant diseases that, in the opinion of the investigator, may influence (due to the disease itself and/or its treatments) the development, evolution, or assessment of the HHT. Thus, the exclusion was made following this statement.

If comparison was made excluding this patient, the mean would then be: 4.14 before versus 2.74 after treatment, with a decrease in the ESS of 1.4.

Most of the cases were from patients with mild epistaxis according to the ESS scale.

Especially remarkable were cases when the epistaxis was more important, moderate in three cases, and severe in one case. In these patients, treatment with etamsylate reduced bleeding by more than 1 point according the ESS scale, and the epistaxis was changing from category to category.

In case 3, epistaxis score changed from 4.57 (moderate) to 1.74 (lower mild), decreasing by 2.83.

In case 1, the score decreased from 7.36 (severe bleeding) to 4.42 (moderate bleeding in the lower range), lowering by 2.94 in the score.

In case 4, the patient was with moderate epistaxis (score of 5.07) and the score decreased to 3.15 (mild), lowering by 2.12 points in the scale.

Finally in patient 8, the score lowered from 5.25 (moderate) to 3.44 (mild), lowering by 1.81 points.

No adverse events were recorded.

Patient 5, with no changes, had mild epistaxis and in this case it was more difficult to record a visible effect in such a short time, 4 weeks.

The authors acknowledge the weaknesses of the clinical trial and the necessary steps are underway to achieve the double-blind randomized, and if possible, multicentric trial. Moreover, a possible placebo effect may be present, and this fact should be corrected in a future clinical trial.

### Statistical Analysis

Student's *t* -test was used to compare mean ESS before and after the treatments in the different comparisons.

- Mean ESS before and after the clinical trial showed significant differences, with *p* < 0.001 (***).
- When the mean ESS was made in the six HHT1 patients comparing before and after the clinical trial, differences were also significant with *p* < 0.01 (**).
- Differences between mean ESS in the six HHT2 patients before and after the treatment did reveal also statistical significance, although with lower a *p* -value, *p* < 0.05 (*).
- There were no significant differences between HHT1 (1.514) and HHT2 (1.296) patients comparing mean ESS before and after the treatment ( *p* -value = 0.719, ns)
- There were no significant differences observed when comparing the responses between males (1.157) and females (1.531). *p* -Value = 0.54; therefore no differences concerning the sex variable (ns) in the response were found.

Analysis of VEGF and FGF, biomarkers related to angiogenesis and bleeding, both parameters related to HHT, were made by commercial ELISA (R&D) in the plasma of the patients before and after the 4 weeks of the clinical trial. The results revealed that etamsylate decreased significantly the levels of FGF (9.13 pre- vs. 7.9 posttreatment) and VEGF (36.15 pre-, vs. 18.84 post, with *p* < 0.001 [***]). VEGF plasma levels decreased in 7 out of 11, while FGF decreased in 9 out of 11. The results are shown in Fig. 7C , D . Friedman's no-parametric median comparisons were used as statistical analysis. The box–whiskers corresponding plots are shown.

## Discussion

There is no specific drug therapy for HHT, and the treatments used are not standard, but rather depend on the experience of physicians from reference centers. Several palliative therapeutic measures have been proposed: transfusion of blood derivatives, infusions of iron for anemia, embolization for pulmonary and brain malformations, and the use of treatments to reduce bleeding, mainly epistaxis.

These treatments include tranexamic acid to strengthen coagulation and SERMs, of which raloxifene and bazedoxifene were designated as orphan medicines by the EMA in 2010 (EMA 10–3099) and 2014 (EMA 14–8862), respectively. The most significant side effect of antifibrinolytics and SERMs are the risk of deep venous thrombosis, in patients with previous clinical history of thrombosis or hypercoagulability. 49 In cases of severe epistaxis, the use of antiangiogenic treatments with bevacizumab and thalidomide has been tested, with temporary and contentious results.

The treatment reported in the present work is novel for nose bleeding in HHT patients, and would belong to the antiangiogenic strategy. It is based on the inhibition of the angiogenic FGF signaling pathways, described in the introductory section, by dobesilate. This compound is described in the pharmacology literature as a product with vasculotropic effects, a nontechnical concept that attempts to bring together several relatively imprecise findings for vascular protection accumulated through many years of medical use. As summarized under the section Introduction, until the year 2010, 17 the mechanism of action of dobesilate was unknown. Inhibition of FGF could essentially explain the vascular protection observed in the regular medical practice. In the case of etamsylate, used for many years to treat diabetic retinopathy and chronic venous insufficiency without reported side effects, it is a drug available as an injectable solution and has been used in this assay as an aerosol.

In HHT, the state of the aberrant/damaged capillary network favors the formation of telangiectases with the consequent susceptibility to bleeding. It has been observed in the clinical trial here reported that the repeated intranasal application (as a spray) of etamsylate to HHT patients with high susceptibility to nose bleeds can ameliorate the condition by the reduction of the telangiectases.

Etamsylate inhibits in vitro angiogenesis of endothelial cells. Although in the cell line of microvasculature HMEC-1, the results were not significant, in the endothelial primary cultures HUVECs or BOECs, the differences were statistically significant. The discrepancy in the results could be derived from the nature of a cell line HMEC-1, which has been artificially immortalized, and whose properties are far from physiologically relevant cells, as in the case of BOECs from HHT patients. This effect would lead to a reduction in the neovessel formation after stress or wounding of nasal mucosa. The antiangiogenic properties of etamsylate were demonstrated in vitro where it was able to inhibit cell migration and Matrigel tubulogenesis especially at the highest doses. Therefore, etamsylate is a candidate for the antiangiogenic strategy in the treatment of HHT-derived epistaxis. Etamsylate treatment of endothelial cells has shown absence of toxicity, in the range of doses used in this study. Since etamsylate binds FGF and in this way interferes with the binding of FGF to its receptor, cells treated with etamsylate show a decreased phosphorylation of Akt and MAPK Erk1/2, in a dose-dependent manner, interfering in this way with the signaling cascades of both, VEGF and FGF.

The first limitation of the clinical trial is the lack of randomization with blinding of patients and researchers, and therefore vulnerability to placebo effects. The low number of patients is a secondary limitation, and would have been a more significant limitation had no statistically significant result been observed. Furthermore, a possible placebo effect may be present and this fact should be corrected in a future clinical trial. Finally, one of patients was withdrawn since he was being treated at the same time with hormones to build body mass. Steroids and other body mass substances are proangiogenic and increase the blood flow; therefore, they are potentially “epistaxis triggering” factors in HHT patients. These substances should be included in the exclusion criteria when recruiting patients in HHT clinical trials. The participants in the clinical trial were HHT patients attending “a consult for rare diseases” at the Ramón y Cajal Hospital in Madrid. This was a pilot trial, the first one made for this disease, in Spain and with this particular drug. On the other hand, the topical application using spray was also a novel way of administering etamsylate, only available in liquid vials for injection.

**Fig. 8:**
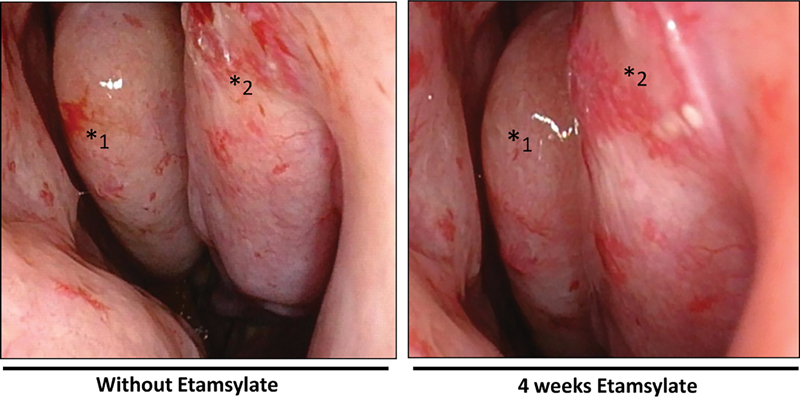
The antiangiogenic effect of etamsylate on the nose mucosa. Aspect of the nose mucosa in a patient before starting the treatment, and around the same area after 4 weeks of treatment with etamsylate (* shows the major affected area).

Moreover, the short time of treatment was a major limitation which may have precluded getting higher improvement, measured as further reduction of epistaxis, in time. The treatment period of observation was 4 weeks. According to the clinical trial observation, the patients started to experience a decrease in time and frequency of bleeding after 3 weeks of treatment, which is in agreement with an underlying mechanism of antiangiogenesis with a progressive reduction of the enlarged vessels (telangiectases) as shown in Fig. 8 . The short time fixed for the clinical trial was really challenging, and corresponded roughly to the minimal time which could reveal significant improvement, in case of success of treatment. The constraint in time was mainly due to economic reasons, as explained later. Etamsylate is not currently sold in liquid vials in Spain. Therefore, we had to import the product from Sanofi-Aventis, France.

The present assay was an independent clinical trial supported by the Spanish HHT Patient Association with public utility declaration (a nongovernmental organization) and our laboratory. The costs were not only for the medicinal product and vials, but also for payment for a CRO (Meditrial) to legally control the clinical trial, according to the current law. On the other hand, insurance had to be paid for the patients and the social responsibility of the researcher, since it was not considered a low intervention clinical trial. All these constraints justified to shorten the time to a limit that was the minimum to get outcomes. The results of the clinical trial together with basic knowledge of the disease and the antiangiogenic in vitro results presented in this work led to the orphan drug designation of etamsylate by the EMA (EU/3/18/2087) in 2018.
